# Vitamin D deficiency as a potential risk factor for thyroid cancer: a clinical perspective

**DOI:** 10.3389/fnut.2025.1645851

**Published:** 2025-10-10

**Authors:** Jing Yang, Tianyu Wu, Xiaoying Chang, Yue Pan, Jian Gong, Chuanjia Yang

**Affiliations:** ^1^Department of Neurology, Shengjing Hospital of China Medical University, Shenyang, Liaoning, China; ^2^Department of General Surgery, Shengjing Hospital of China Medical University, Shenyang, Liaoning, China; ^3^Department of Pathology, Shengjing Hospital of China Medical University, Shenyang, Liaoning, China; ^4^Department of Laboratory Medicine, Shengjing Hospital of China Medical University, Shenyang, Liaoning, China; ^5^Department of Clinical Pharmacy, School of Life Science and Biopharmaceutics, Shenyang Pharmaceutical University, Shenyang, China

**Keywords:** vitamin D deficiency, thyroid cancer, thyroidectomy, hypocalcemia, hypothyroidism

## Abstract

**Objectives:**

Vitamin D deficiency has garnered increasing attention as a potential risk factor for thyroid dysfunction and oncological progression. This study was designed to investigate the relationship between preoperative vitamin D status and postoperative complications in patients undergoing thyroidectomy.

**Methods:**

This prospective cohort study investigated the association between preoperative vitamin D status and postoperative outcomes in a cohort of 120 patients following thyroidectomy for benign or malignant thyroid disease at Shengjing Hospital, China (2020–2022). Participants were stratified into three groups based on serum 25-hydroxyvitamin D [25(OH)D] concentrations: deficient (<20 ng/mL), insufficient (20–30 ng/mL), and sufficient (>30 ng/mL).

**Results:**

Vitamin D-deficient patients exhibited higher preoperative TSH levels (4.8 ± 1.6 vs. 2.7 ± 0.9 mIU/L, *p* < 0.001), lower free T3/T4, and reduced serum calcium (8.5 ± 0.6 vs. 9.1 ± 0.5 mg/dL, *p* = 0.004) compared to sufficient patients. After operation, vitamin D deficiency was associated with increased odds of hypocalcemia (OR: 4.17, 95%CI: 1.31–13.35, *p* = 0.01) and hypothyroidism (OR = 2.91, 95%CI: 1.14–7.42, *p* = 0.02) after adjustment for potential confounders.

**Conclusion:**

The findings of this study indicate that preoperative screening for, and subsequent correction of, vitamin D deficiency could lead to improved postoperative recovery and a reduction in complications among patients undergoing thyroidectomy. Further research is needed to establish causal relationships and explore the underlying mechanisms linking vitamin D status to thyroid function and surgical outcomes.

## Introduction

Thyroid cancer is the most prevalent endocrine malignancy, with a global incidence that has markedly increased over the past few decades (1). Although this rising incidence is attributable to a combination of factors—such as enhanced diagnostic imaging capabilities and the potential for overdiagnosis—a true increase in certain demographic groups points to the role of modifiable risk factors, including those related to environment and nutrition (2). Among these, vitamin D deficiency has emerged as a potential contributor to thyroid carcinogenesis, based on its immunomodulatory, antiproliferative, and pro-differentiation properties (3, 4).

Vitamin D is a steroid hormone primarily synthesized in the skin upon exposure to ultraviolet B radiation and subsequently activated in the liver and kidneys (5). Beyond its classical role in calcium-phosphorus homeostasis, vitamin D regulates cell cycle, apoptosis, and immune surveillance—all critical pathways in cancer biology (6). The vitamin D receptor (VDR), expressed in thyroid tissue, modulates the transcription of genes involved in cellular proliferation and differentiation (7). Altered VDR expression in thyroid tumors supports a functional role of vitamin D signaling in thyroid pathology (8, 9).

Epidemiological data linking vitamin D status with thyroid cancer risk remain inconclusive. A number of studies have reported markedly reduced serum concentrations of 25-hydroxyvitamin D [25(OH)D] in individuals with hypothyroidism, autoimmune thyroid disorders, and Hashimoto’s thyroiditis relative to healthy control subjects (10, 11), while others find no significant association (12, 13). These inconsistencies may stem from variations in study design, geographic differences in sunlight exposure, and confounding factors like obesity and autoimmune thyroid disease (14). Nevertheless, vitamin D deficiency may correlate with aggressive disease features and poorer outcomes (15). Clinically, vitamin D deficiency exacerbates postoperative complications in thyroid cancer patients. Hypocalcemia and hypothyroidism following thyroidectomy are more prevalent and severe in vitamin D-deficient individuals due to impaired parathyroid function and altered thyroid hormone dynamics (15, 16). Emerging evidence suggests vitamin D status may also influence long-term recurrence risk during TSH suppression therapy (17–19).

Given these knowledge gaps, this study investigates the association between preoperative vitamin D status and postoperative outcomes in thyroidectomy patients. By analyzing a well-characterized cohort stratified by serum 25(OH)D levels, we aim to clarify vitamin D’s prognostic role in thyroid cancer management.

## Methods

This study enrolled 458 patients diagnosed with thyroid cancer who underwent either radical or expanded radical thyroidectomy at the Department of General Surgery, Shengjing Hospital of China Medical University, between June 2020 and June 2022. The included participants were diagnosed with benign thyroid tumors and papillary thyroid carcinoma by pathological histology. The participants also excluded from the study if they: (A) did not have available data on vitamin D intake or serum 25(OH)D levels, (B) received vitamin D supplementation before and after the surgery, (C) had a history of other cancers at the time of disease diagnosis, (D) diagnosed with the history of conditions that cause hypercalcemia or hypercalciuria (e.g., kidney diseases, bone diseases, and multiple myeloma), and (E) received medications that may affect serum calcium or vitamin D levels. All study subjects signed an informed consent form before participation. Of the initial cohort, 120 participants who met the predefined inclusion criteria and were aligned with the study objectives were selected for the final analysis.

The study protocol was approved by the ethics scientific committee of the Shengjing Hospital (ethical code number: M0731) and all participants filled in an informed consent form before participating in the study. Also, the study followed the ethical principles outlined in the Declaration of Helsinki.

### Assessment of the demographic information and the outcomes

Demographic characteristics, including age, gender, medical history, city, height, and weight of the study subjects, were collected. Pre-and postoperative venous blood samples (5 mL each) were collected from all participants. Serum concentrations of T3, T4, TSH, TPOAb, TgAb, calcium, phosphorus, and parathyroid hormone (PTH) were quantified using chemiluminescent immunoassay. All study subjects underwent ultrasound examination of the thyroid gland, and the size and number of tumors and the presence of lymph node metastases were recorded. An experienced ultra-sonographer reported the thyroid ultrasound results. Moreover, serum 25-(OH) D3 levels were measured by Cobas 8,000 automatic biochemical analyzers before and after the surgery. The levels of serum 25-(OH) D3 were predefined as sufficient (>30 ng/mL), insufficient (20–30 ng/mL), and deficient (<20 ng/mL). Serum calcium was recorded in mmol/L; hypocalcemia was defined as <2.10 mmol/L.

### Statistical analysis

The findings were presented as mean ± SD for quantitative and number (%) for qualitative variables. Paired *t*-test or Wilcoxon signed-rank test was used for comparison of quantitative variables before and after the operation. Continuous variables were compared across vitamin D (VD) status categories (deficient <20 ng/mL; insufficient 20–30 ng/mL; sufficient >30 ng/mL) using one-way ANOVA or Kruskal–Wallis tests as appropriate. When omnibus tests were significant, Bonferroni-adjusted *post hoc* pairwise comparisons were conducted. Categorical variables were compared using *χ*^2^ or Fisher’s exact tests. For primary outcomes (hypocalcemia, hospital stay >3d, and hypothyroidism), we additionally fitted multivariable logistic regression models with VD status as the main exposure and age, sex, smoking status, diabetes, hypertension, vitamin D supplementation history, season of blood sampling, and BMI as covariates (VD-sufficient group served as the reference). We report adjusted odds ratios (ORs) with 95% confidence intervals (CIs) and two-sided *p*-values. Analyses were performed in SPSS 26.0/GraphPad Prism 7.

## Results

Table 1 presents the demographic, biochemical, and clinical characteristics of participants stratified by serum vitamin D (VD) levels before operation. No statistically significant differences were observed in age (*p* = 0.08) or body mass index (BMI) (*p* = 0.22) across the three groups. However, significant differences were noted in several thyroid and biochemical parameters: TSH levels progressively decreased with higher vitamin D status, with the highest mean TSH observed in the deficient group (4.8 ± 1.6 mIU/L) and the lowest in the sufficient group (2.7 ± 0.9 mIU/L; *p* = 0.001). FT3 and FT4 levels were significantly elevated in participants with sufficient vitamin D levels (FT3: *p* = 0.02; FT4: *p* = 0.03). Serum calcium concentrations were lowest among VD-deficient participants (8.5 ± 0.6 mg/dL), showing a significant upward trend with improved VD status (*p* = 0.004). Although differences in qualitative variables did not reach statistical significance, clinically relevant trends were observed: TPO antibody positivity was more common in the VD-deficient group (34%) compared to the VD-sufficient group (18%; *p* = 0.15). Also, Smoking prevalence was higher among VD-deficient individuals (29%) versus those with sufficient VD levels (11%; *p* = 0.12).

**Table 1:** Demographic, biochemical, and clinical characteristics of participants before operation.

Variable	Deficiency (VD < 20 ng/mL) (*n* = 35)	Insufficiency (20 ≤ VD ≤ 30 ng/mL) (*n* = 40)	Sufficiency (VD > 30 ng/mL) (*n* = 45)	*p*-value*
Age (years)	45.2 ± 12.3	48.5 ± 10.7	50.1 ± 9.8	0.08
BMI (kg/m^2^)	26.1 ± 3.5	27.3 ± 4.1	26.8 ± 3.8	0.22
TSH (mIU/L)	4.8 ± 1.6	3.9 ± 1.2	2.7 ± 0.9	0.001
FT3 (pg/mL)	1.1 ± 0.4	1.3 ± 0.3	1.5 ± 0.2	0.02
FT4 (ng/dL)	0.9 ± 0.2	1.0 ± 0.3	1.2 ± 0.2	0.03
Calcium (mg/dL)	8.5 ± 0.6	9.1 ± 0.5	9.4 ± 0.4	0.004
Gender				0.54
Female	20 (57%)	25 (62%)	30 (67%)	
Male	15 (43%)	15 (38%)	15 (33%)	
Smoking status				0.12
Smoker	10 (29%)	8 (20%)	5 (11%)	
Non-smoker	25 (71%)	32 (80%)	40 (89%)	
TPO antibody				0.15
Positive	12 (34%)	10 (25%)	8 (18%)	
Negative	23 (66%)	30 (75%)	37 (82%)	
Tumor type				0.31
Papillary	28 (80%)	35 (88%)	36 (80%)	
Follicular	7 (20%)	5 (12%)	9 (20%)	
Comorbidities				0.45
Diabetes	8 (23%)	6 (15%)	5 (11%)	
Hypertension	12 (34%)	10 (25%)	9 (20%)	

Data are presented as mean ± SD and number (%).

*From ANOVA or Kruskal–Wallis tests for quantitative variables and *χ*^2^ or fisher exact tests for qualitative variables.

Table 2 summarizes the longitudinal changes in thyroid function markers and relevant postoperative outcomes. After the operation, TSH levels declined significantly across all VD groups, with the greatest reduction observed in VD-deficient patients (Δ = −2.9 mIU/L, *p* < 0.001). FT3 and FT4 levels significantly increased in the VD-deficient and insufficient groups (*p* < 0.05), while the increase in the sufficient group was smaller and not significant for FT3 (*p* = 0.08). Serum calcium levels decreased postoperatively in all groups, with the largest decline in the VD-deficient group (Δ = −0.3 mg/dL; *p* = 0.005). Postoperative clinical outcomes further underscored the role of VD status: Hypocalcemia occurred significantly more frequently in VD-deficient patients (34.3%) compared to VD-sufficient individuals (11.1%; *p* = 0.03). Postoperative hypothyroidism was more prevalent in VD-deficient participants (51.4%) versus those with VD sufficiency (26.7%; *p* = 0.04). Although not statistically significant, prolonged hospitalization (>3 days) was more common among VD-deficient patients (42.9%) compared to the sufficient group (22.2%; *p* = 0.12).

**Table 2:** Longitudinal changes in thyroid function markers and relevant postoperative outcomes.

Outcome	Vitamin D group	Pre-op	Post-op	*p*-value*
TSH (mIU/L)	<20	4.8 ± 1.6	2.1 ± 0.8	<0.001
	20–30	3.9 ± 1.2	1.9 ± 0.6	<0.001
	>30	2.7 ± 0.9	1.5 ± 0.4	0.003
FT3 (pg/mL)	<20	1.1 ± 0.4	1.5 ± 0.3	0.006
	20–30	1.3 ± 0.3	1.6 ± 0.2	0.01
	>30	1.5 ± 0.2	1.7 ± 0.2	0.08
FT4 (ng/dL)	Deficient	0.9 ± 0.2	1.2 ± 0.3	0.01
	Insufficient	1.0 ± 0.3	1.3 ± 0.2	0.003
	Sufficient	1.2 ± 0.2	1.4 ± 0.2	0.02
Calcium (mg/dL)	Deficient	8.5 ± 0.6	8.2 ± 0.7	0.005
	Insufficient	9.1 ± 0.5	8.9 ± 0.6	0.01
	Sufficient	9.4 ± 0.4	9.3 ± 0.5	0.15

Variable	VD < 20 ng/mL (*n* = 35)	20 ≤ VD ≤ 30 ng/mL (*n* = 40)	VD > 30 ng/mL (*n* = 45)	*p*-value*
Post-op hypocalcemia	12 (34.3%)	8 (20.0%)	5 (11.1%)	0.02
TPO Ab+ → post-op	8/12 (66.7%)	5/10 (50.0%)	3/8 (37.5%)	0.33
Hospital stay >3d	15 (42.9%)	12 (30.0%)	10 (22.2%)	0.15
Hypothyroidism	18/35 (51.4%)	15/40 (37.5%)	12/45 (26.7%)	0.04

Data are presented as mean ± SD and number (%).

*From paired *t*-test or Wilcoxon signed-rank test for quantitative variables and *χ*^2^ and fisher exact tests for qualitative variables.

**Figure 1:**
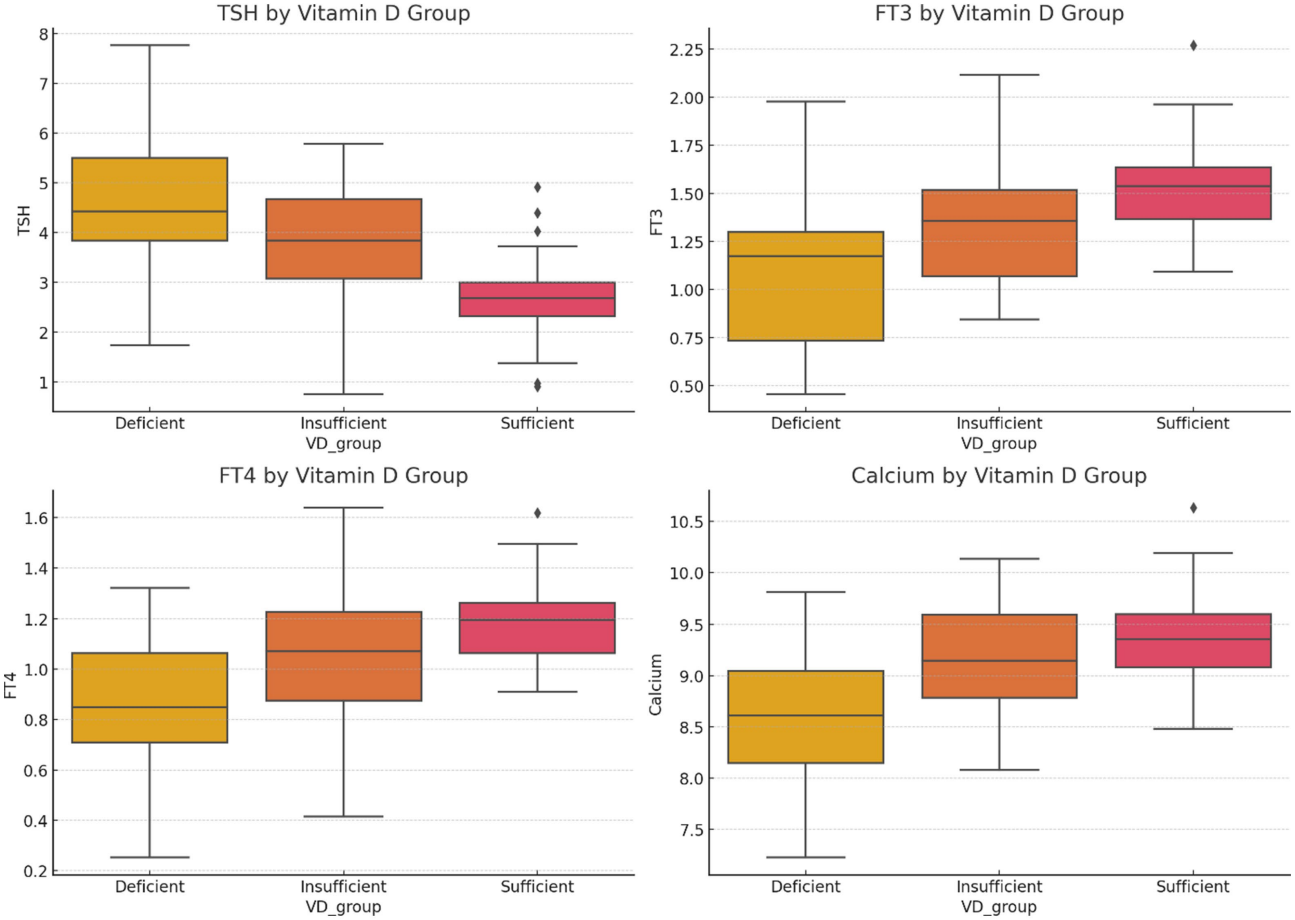
Boxplots of TSH, FT3, FT4, and serum calcium levels across VD status groups (deficient, insufficient, sufficient) illustrate clear trends.

**Figure 2:**
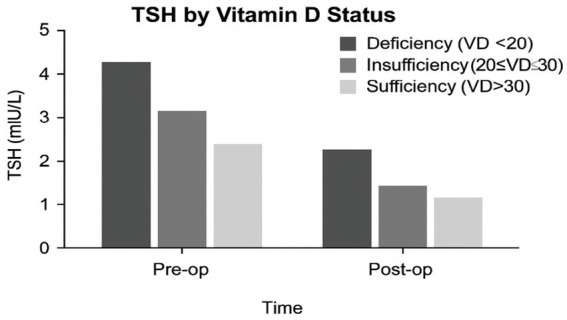
TSH levels by vitamin D status at pre-and postoperative time points.

The levels of TSH, FT3, FT4, and serum calcium across VD status groups (deficient, insufficient, and sufficient) are shown in Figure 1. As seen, TSH levels are lowest in VD-sufficient individuals, T3 and FT4 levels rise with higher VD levels, and serum calcium shows a positive association with VD status. In Figure 2, mean TSH values before and after surgery by the VD group are compared. Accordingly, the VD Deficient participants (VD < 20 ng/mL) had the highest preoperative TSH (~4.3 mIU/L), with a substantial postoperative drop (~2.3 mIU/L). In VD, insufficient participants (20–30 ng/mL), we found moderate preoperative TSH (~3.1 mIU/L), with a substantial postoperative drop to ~1.6 mIU/L. For VD, sufficient participants (VD > 30 ng/mL), the lowest baseline TSH (~2.5 mIU/L) and smallest postoperative reduction (~1.2 mIU/L) have been found.

Table 3 presents the results of multivariable logistic regression analyses examining the association between preoperative vitamin D status and postoperative outcomes, adjusted for age, sex, smoking status, diabetes, hypertension, history of vitamin D supplementation, season of blood sampling, and BMI. Vitamin D deficiency (<20 ng/mL) was significantly associated with an increased risk of postoperative hypocalcemia (OR = 4.17, 95% CI 1.31–13.35; *p* = 0.01) and hypothyroidism (OR = 2.91, 95% CI 1.14–7.42; *p* = 0.02) compared with patients with sufficient patients. Those with insufficient vitamin D levels (20–30 ng/mL) showed a nonsignificant trend toward higher odds of hypocalcemia (OR = 2.00, 95% CI 0.66–6.71; *p* = 0.26) and hypothyroidism (OR = 1.65, 95% CI 0.66–4.14; *p* = 0.28). Prolonged hospitalization (>3 days) was more frequent among vitamin-deficient patients (OR = 2.63, 95% CI 0.99–6.93) and those with insufficient vitamin D (OR = 1.50, 95% CI 0.57–3.80), although these associations did not reach statistical significance (*p* = 0.05 and *p* = 0.41, respectively).

**Table 3:** Multivariable logistic regression of postoperative hypocalcemia, hospital stay, and hypothyroidism by vitamin D status.

Variables	VD < 20 ng/mL (*n* = 35)	20 ≤ VD ≤30 ng/mL (*n* = 40)	VD > 30 ng/mL (*n* = 45)
	OR (95%CI), *p*-value	OR (95%CI), *p*-value	
Hypocalcemia			
Crude	5.45 (1.64–12.54), 0.005	3.25 (0.77–5.64), 0.14	1
Adjusted model	4.17 (1.31–13.35), 0.01	2.00 (0.66–6.71), 0.26	1
Hospital stay >3d			
Crude	3.42 (0.84–5.75), 0.35	2.45 (0.67–4.75), 0.23	1
Adjusted model	2.63 (0.99–6.93), 0.51	1.50 (0.57–3.81), 0.41	1
Hypothyroidism			
Crude	2.75 (1.56–6.78), 0.003	1.56 (0.45–4.56), 0.35	1
Adjusted model	2.91 (1.14–7.42), 0.02	1.65 (0.66–4.14), 0.28	1

VD, vitamin D.

The analyses were adjusted for age, sex, smoking status, diabetes, hypertension, vitamin D supplementation history and season of blood sampling and BMI.

Reference category: VD sufficient (>30 ng/mL).

## Discussion

The findings of this study indicate that vitamin D deficiency is an independent predictor of both postoperative hypocalcemia and hypothyroidism, whereas its association with extended hospital stay appears weaker. Vitamin D (VD) has long been recognized for its role in bone health and calcium metabolism, but emerging evidence suggests that it also has a significant impact on thyroid function. The findings of this study underscore the potential association between vitamin D status and thyroid-related parameters such as TSH, FT3, FT4, and postoperative outcomes following thyroidectomy. This finding highlights the clinical implications for preoperative management of vitamin D deficiency in patients undergoing thyroid surgery.

The results of this investigation demonstrate a clear association between vitamin D deficiency and altered thyroid function, manifesting as significantly elevated thyroid-stimulating hormone (TSH) levels alongside reduced concentrations of free triiodothyronine (FT3) and free thyroxine (FT4). These findings are consistent with a growing body of evidence from previous research examining the interplay between vitamin D status and thyroid hormone regulation. Previous research has shown that low levels of vitamin D are often accompanied by altered thyroid function, including elevated TSH levels (20, 21). These findings are consistent with earlier studies that have reported an inverse relationship between vitamin D and TSH, suggesting that vitamin D may play a role in modulating thyroid hormone synthesis (22).

Vitamin D has been proposed to exert an effect on thyroid hormone production through its interaction with the vitamin D receptor (VDR) expressed in thyroid tissue (23). It has been suggested that vitamin D may influence thyroid function by regulating gene expression and affecting thyroid cell proliferation (24). Moreover, vitamin D is thought to modulate the synthesis and metabolism of thyroid hormones by influencing the deiodinase enzymes, which convert T4 to the active form of T3 (25). These molecular mechanisms may explain the observed elevation of FT3 and FT4 levels in individuals with sufficient vitamin D status.

The well-established role of vitamin D in regulating calcium metabolism is further supported by the present study’s results, which demonstrated that the lowest serum calcium concentrations were observed in the cohort with vitamin D deficiency. Calcium homeostasis is one of the key functions regulated by vitamin D, and deficiency can lead to hypocalcemia (26). In the current study, the significant trend of increasing serum calcium with improved vitamin D status highlights the importance of vitamin D in maintaining proper calcium levels, especially in the perioperative period following thyroidectomy.

The postoperative decline in calcium levels observed in all groups, but most notably in the VD-deficient group, underscores the risk of hypocalcemia after thyroid surgery. This is consistent with other studies that have shown that vitamin D deficiency is a known risk factor for postoperative hypocalcemia, which is a common complication following thyroidectomy due to potential damage to the parathyroid glands (27). The significantly higher rates of hypocalcemia in the VD-deficient group compared to those with sufficient vitamin D further suggest that vitamin D status should be considered a modifiable risk factor for postoperative complications (28, 29). While the mean postoperative decrease in serum calcium in the deficient group (Δ = −0.3 mg/dL) may appear modest, its clinical impact is substantial in the context of thyroidectomy. The absolute postoperative calcium level is a critical determinant of symptoms. The deficient group’s levels fell from an already lower baseline (8.5 ± 0.6 mg/dL) to a postoperative mean of 8.2 ± 0.7 mg/dL. This places a significant proportion of these patients near or below the threshold for biochemical hypocalcemia (2.10 mmol/L or 8.4 mg/dL) and into a range where clinical symptoms such as paresthesia, muscle cramps, and Chvostek’s sign are likely to manifest (30). This is directly corroborated by our clinical outcome data, which show a significantly higher incidence of clinically diagnosed hypocalcemia in the deficient group (34.3%) compared to the sufficient group (11.1%; *p* = 0.03). Therefore, the Δ calcium of −0.3 mg/dL is not just a statistically significant finding but represents a physiologically and clinically important decline that translates into a higher risk of symptomatic complications, increased monitoring needs, and the necessity for calcium and vitamin D supplementation postoperatively.

One of the key findings of this study is the higher prevalence of postoperative hypothyroidism in the VD-deficient group (51.4%) compared to the sufficient group (26.7%). This is consistent with several studies that have reported a higher incidence of hypothyroidism in individuals with vitamin D deficiency, particularly after thyroid surgery (28, 31). Vitamin D is thought to influence thyroid function by regulating immune responses, which may help reduce the risk of autoimmune thyroid diseases, such as Hashimoto’s thyroiditis, which is a known cause of hypothyroidism (19, 32, 33). Moreover, studies have shown that vitamin D deficiency can exacerbate autoimmune conditions, including autoimmune thyroiditis, by promoting inflammatory cytokine release and immune cell activation (34). These effects could contribute to the higher rates of postoperative hypothyroidism observed in the current study, as patients with low vitamin D levels may have an impaired ability to recover normal thyroid function after surgery.

Although the study did not find a statistically significant association between vitamin D deficiency and prolonged hospitalization, the trend toward longer stays in the VD-deficient group (42.9% vs. 22.2% in the sufficient group) is noteworthy. Previous research has suggested that vitamin D deficiency is associated with poorer overall health outcomes, including delayed recovery and increased risk of complications (35). Several studies have shown that vitamin D deficiency can impair immune function, increase susceptibility to infections, and delay wound healing, all of which could contribute to prolonged hospitalization (36). While the current study did not find a statistically significant association with prolonged hospitalization, the higher percentage of patients with extended stays in the VD-deficient group warrants further exploration in larger, longitudinal studies to confirm the potential impact of vitamin D on recovery after thyroidectomy.

The findings from this study suggest that vitamin D deficiency is an important factor to consider in the preoperative evaluation of patients undergoing thyroid surgery. Given the observed associations between vitamin D deficiency and increased risk of postoperative hypocalcemia, hypothyroidism, and prolonged recovery, preoperative screening and correction of vitamin D deficiency may improve surgical outcomes. This is consistent with the recommendations of several studies and expert guidelines that advocate for the correction of vitamin D deficiency in patients undergoing various surgical procedures (37). Supplementing with vitamin D prior to surgery has been shown to reduce the incidence of postoperative complications, including hypocalcemia and delayed recovery (38). Given the significant role of vitamin D in both calcium homeostasis and immune function, optimizing vitamin D levels before thyroid surgery could enhance recovery, reduce complications, and potentially shorten hospitalization. The limited sample size directly reduces the statistical power of our analyses, increasing the risk of Type II errors (i.e., failing to detect a true effect where one exists). This is particularly relevant for outcomes that showed strong clinical trends but did not reach conventional statistical significance, most notably the duration of hospitalization. We observed that 42.9% of deficient patients had a prolonged hospital stay (>3 days) compared to 22.2% in the sufficient group (*p* = 0.12). The multivariable analysis also suggested a strong association (OR = 2.63, 95% CI: 0.99–6.93; *p* = 0.05), which was on the precise borderline of significance. Also, it is highly plausible that with a larger cohort, this clinically meaningful difference would have achieved statistical significance. Similarly, the higher prevalence of TPO antibody positivity and smoking in the deficient group, while not statistically significant in this sample (*p* = 0.15 and *p* = 0.12, respectively), may represent true associations that our study was underpowered to confirm.

Despite the important findings of this study, there are several limitations that should be addressed in future research. First, a single-center setting can lead to possible referral bias. Second, due to the absence of long-term outcomes (recurrence, survival), we could not check the association for the long term. Third, the study did not assess the underlying mechanisms through which vitamin D affects thyroid function. Future research should focus on investigating the molecular pathways involved in vitamin D’s regulatory effects on the thyroid gland and calcium metabolism. Fourth, although we controlled the analyses for possible confounders, other possible residual confounding needs to be considered. Fifth, larger sample size studies are needed to validate these findings and determine the optimal vitamin D levels required for maintaining thyroid health and improving surgical outcomes. Finally, while the data support an association between vitamin D deficiency and adverse outcomes, causal claims should be toned down. The discussion should emphasize that this is an observational association, and randomized controlled trials are needed to confirm the benefit of supplementation.

In conclusion, this study provides valuable insights into the impact of vitamin D status on thyroid function and postoperative outcomes following thyroidectomy. The results suggest that vitamin D deficiency is associated with higher TSH levels, lower FT3 and FT4 levels, and increased risk of hypocalcemia and hypothyroidism after surgery. These findings highlight the importance of monitoring and correcting vitamin D deficiency in the preoperative setting to improve patient outcomes. Future research should further explore the molecular mechanisms linking vitamin D to thyroid function and investigate the potential benefits of preoperative vitamin D supplementation.

## Data Availability

The raw data supporting the conclusions of this article will be made available by the authors, without undue reservation.
